# Gene regulatory logic of the interferon-β enhancer is characterized by two selectively deployed modes of transcription factor synergy

**DOI:** 10.1073/pnas.2502800122

**Published:** 2025-08-12

**Authors:** Allison Schiffman, Zhang Cheng, Diana Ourthiague, Alexander Hoffmann

**Affiliations:** ^a^Signaling Systems Laboratory, Department of Microbiology, Immunology and Molecular Genetics, and the Institute for Quantitative and Computational Biosciences, University of California Los Angeles, CA 90095

**Keywords:** interferon-β, boolean logic gates, thermodynamic state ensemble models, NFκB, interferon regulatory factors (IRF)

## Abstract

Type I interferon IFNβ is a key immune response cytokine, and when its expression is dysregulated, it causes disease. The regulation of IFNβ enhancer has been a touchpoint of mammalian gene control research since the discovery of functional synergy between two stimulus-responsive transcription factors (TFs), nuclear factor kappa B (NFκB) and interferon regulatory factors (IRF). However, subsequent gene knockout studies revealed that in some conditions either NFκB or IRF activation can be dispensable, leaving the precise regulatory logic of IFNβ transcription an open question. Here, we developed a series of quantitative enhancer states models of IFNβ expression control and evaluated them with stimulus–response data from TF knockouts. Of these, our analysis reveals that two modes of TF synergy account for the available data and neither is based on binding cooperativity. The first involves two adjacent IRF dimers, with a sigmoidal binding curve at the distal site rendering it ultrasensitive and restricting it to conditions of high IRF activity upon viral infection. The second is driven by the proximal site, which has high affinity and synergizes with NFκB to enable about half-maximal expression in response to bacterial exposure. Its accessibility is controlled by the competitive repressor p50:p50, which prevents basal IRF from binding, such that NFκB-only stimuli do not induce IFNβ expression and may allow for prior-exposure-dependent tuning. The model explains how the regulatory logic of the IFNβ enhancer ensures invariant IFNβ expression in response to viral exposure, while providing tunable, context-dependent expression in response to bacterial exposure.

## Significance

Precise regulation of the immune cytokine IFNβ is essential for human health. Classic studies established that the transcription factors NFκB and IRF function synergistically in activating IFNβ expression. However, more recent studies revealed that either factor may be dispensable in certain conditions, leaving the regulatory logic of IFNβ transcription an open question. Here, we evaluated several quantitative models to determine what regulatory logic can account for the available data. We found that the enhancer exhibits two synergy modes with specific transcription capabilities that are deployed in a stimulus-specific manner. Specificity is generated by sigmoidal IRF–DNA binding rather than IRF-NFκB cooperativity. The resulting IFNβ regulatory logic model overcomes a bottleneck in predictive modeling the control of innate immune responses.

Type I interferons are key regulators of the innate immune response. The most prominent type I interferon, IFNβ, functions both as an autocrine and paracrine cytokine that has important roles in myriad aspects of health and disease. Its primary role is to activate the expression of antiviral genes (1–3) but it also has broad roles in the adaptive immune response by regulating inflammation (4), and the functions of antigen-presenting cells (5, 6), T cells, B cells, and NK cells (7, 8). Conversely, IFNβ dysregulation is associated with diseases (9, 10), as it can lead to systemic activation of the immune response (11, 12), contribute to immunosuppression (2), and impair cell growth and health by downregulating protein synthesis (3, 13). Consequently, IFNβ expression must be tightly tuned to the specific context and trigger stimulus such that it is expressed only when and to the degree needed to overcome the immune threat.

The key regulatory step that controls IFNβ production is the initiation of transcription of the *ifnb1* gene. Its stimulus response is primarily controlled by one κB site, which binds nuclear factor kappa-light-chain-enhancer of activated B cells (NFκB) and two IRE sites, which bind interferon regulatory factors (IRFs). NFκB and IRF are families of stimulus-responsive transcription factors (TFs) which respond to pathogen-associated molecular patterns (PAMPs) via pathogen recognition receptors (PRRs). Indeed, while PRRs are diverse and encompass toll-like receptors (TLRs), Nod-like receptors (NLRs), Rig-I-like receptors (RLRs), and cytosolic DNA sensing receptors (cGAS) (14, 15), they all converge on the activation of NFκB and IRF signaling via their respective MyD88, TRIF, RIPK2, MAVS, and STING adaptors (16–19).

Foundational studies of the IFNβ enhancer reported functional synergy between NFκB and IRF, i.e., that more IFNβ is expressed when both NFκB and IRF are activated or transfected than the sum of transcription with either factor alone (20, 21). Given the involvement of a chromatin structural protein HMG1 in bending the DNA, these findings led to a proposed model of an IFNβ “enhanceosome” (20), which recruits CBP (22) and requires the concerted activity of NFκB and IRF to recruit remodeling factors to move a nucleosome blocking the transcription start site (23). The IFNβ enhanceosome has been commonly understood to require two IRFs and NFκB to be bound to the enhancer for maximal transcriptional activation of IFNβ (20, 24, 25).

However, this model does not explain the regulation of IFNβ in all conditions. While IRF knockouts confirmed that IFNβ expression is dependent on IRF regardless of the PAMP stimulus or pathogen exposure (26–29), basal IRF may be sufficient in deregulated NFκB system conditions (30). Further, while dependence on NFκB was found upon stimulation with TLR4-inducing LPS (31, 32), NFκB is not required for IFNβ expression upon stimulation with TLR3- and RLR-inducing PolyIC or infection with Sendai virus (27, 32–34). Thus, it remains unclear what regulatory logic governs expression of this key innate immune-coordinating cytokine.

Here we developed a quantitative understanding of the regulatory logic by which IRF and NFκB govern IFNβ expression by evaluating alternate mathematical models of IFNβ regulatory enhancer states with datasets from a variety of stimulus and knockout conditions. We found that multiple synergy modes, each subject to particular dose–response relationships, are accessed in a condition-dependent manner to provide versatile but precise control of the IFNβ enhancer.

## Results

### Defining a Minimal Combinatorial States Model for IFNβ.

**Fig. 1.**
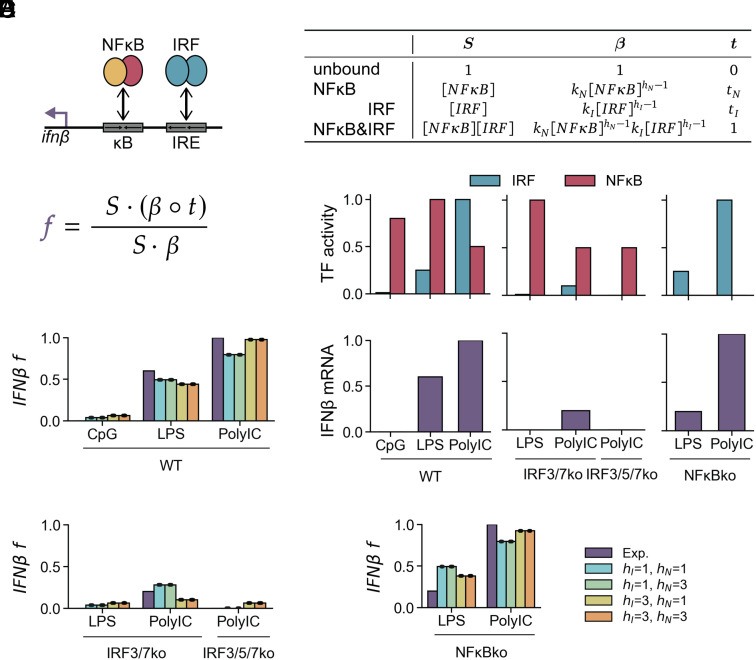
A two-site model does not account for the stimulus-specific NFκB requirement. (*A*) Schematic of IFNβ enhancer with two binding sites, one for each NFκB and IRF, with corresponding binding affinities. Function *f* for calculating IFNβ enhancer activity, where ∘ denotes elementwise multiplication, and variables are defined in (*B*). (*B*) Table of state weights (S), binding equilibrium constants (β), and transcriptional activity (t) for each state of the enhancer. (*C*) Experimental data showing max-normalized activities (MNU) of NFκB and IRF and max-normalized expression of IFNβ mRNA. (*D*–*F*) Experimental and simulated IFNβ expression for four models of $h_{I}$ and $h_{N}$, in (*D*) WT, (*E*) IRF knockouts, (*F*) NFκB knockouts. Dots show predicted expression of best 20 optimized parameter sets, and bars show mean.

To dissect the regulatory logic of the IFNβ enhancer, we used a combinatorial state ensemble model, which allows clear delineation of distinct regulatory modes (35, 36), to calculate IFNβ transcriptional activity f. To evaluate the proposed NFκB-IRF synergy, we first modeled only two binding sites: one for NFκB and one for IRF (Fig. 1*A* and *SI Appendix*, *SI Methods*). The resulting two-site state ensemble model has four enhancer states: unbound, bound to NFκB, bound to IRF, or bound to both NFκB&IRF. The activities of the corresponding proteins in max-normalized units (MNU) for each state can be described by the functional state vector S and the transcriptional activity of each state by the vector t. The unbound state has no transcriptional activity ($t_{0}=0$) and the NFκB&IRF state has maximal transcriptional activity ($t_{\mathrm{IN}}=1$), while the IRF state and the NFκB state have unknown transcriptional activities ($t_{I}$ and $t_{N}$, respectively). Each site has a binding equilibrium constant for its cognate protein, together comprising the vector β. Given the multivalent DNA–protein interaction surfaces and complex chromatinized state of DNA in the nucleus, transcription factors binding to DNA do not always conform to mass action binding kinetics (37). The resulting nonlinear binding curves may be approximated by Hill coefficients (38, 39). To account for this possibility, we let the binding equilibrium for IRF and NFκB be $k_{I}{\left[IRF\right]}^{h_{I}-1}$ and $k_{N}{\left[NF\kappa B\right]}^{h_{N}-1}$, respectively, where $h_{I}$ and $h_{N}$ are Hill coefficients that determine the linearity of binding to their cognate sequences. If $h_{I}=1$ or $h_{N}=1$, then the respective dimer binds linearly; if it is >1, then binding is nonlinear, resulting in ultrasensitivity. The promoter activity (f) is calculated from the S, t, and β vectors (Fig. 1*B*).

To fit the model, we surveyed literature data on IFNβ regulation to generate a data matrix (Fig. 1*C* and *SI Appendix*, Table S1) quantifying response to double-stranded CpG DNA, which activates NFκB but almost no IRF and stimulates no detectable IFNβ (16, 30), bacterial membrane component LPS, which induces low IRF and maximal NFκB and stimulates a medium amount of IFNβ expression (26, 31, 32, 40, 41), and viral analog PolyIC, which induces maximal IRF and stimulates maximal IFNβ expression (16, 26, 27, 29, 32, 42) in WT. We also had data for four genetic knockouts. IRF3/5/7ko cells with PolyIC stimulus produce no detectable IFNβ, while IRF3/7ko cells with PolyIC stimulus produce a little IFNβ, though the weaker IRF-inducing stimulus LPS shows no IFNβ induction even in this double ko (26–29). In contrast, NFκBko cells, which lack subunits RelA and cRel, show severely diminished IFNβ expression when LPS is the stimulus, while they show no decrease in IFNβ expression from WT when PolyIC is the stimulus (27, 31–34).

To determine the regulatory logic that best explains the experimental data, we fit the model parameters to the available data (Fig. 1*C*) and selected the top 20 optimized parameter sets (*SI Methods*), which all gave essentially the same predicted values for IFNβ f. All models correctly predict IFNβ expression in WT cells (Fig. 1*D*) and IRF knockouts (Fig. 1*E*), though models where $h_{I}=1$ somewhat underpredict IFNβ expression in PolyIC-stimulated WT cells. However, all models fail to correctly predict the deficiencies in IFNβ activation in NFκB knockout cells stimulated with LPS (Fig. 1*F*). A high $h_{I}$ allows for more NFκB dependence in response to LPS than in response to PolyIC, but not to the extent observed experimentally and at the expense of LPS-inducibility in WT cells (Fig. 1*D*). The value of $h_{N}$ does not appear to affect the fit of any condition (Fig. 1 *D*–*F*).

Although IRF and NFκB do not show binding cooperativity in vitro (43), both contact CBP/p300 (44, 45), which could in principle mediate such binding cooperativity in vivo. We therefore tested alternative models where IRF and NFκB have binding cooperativity (*SI Appendix*, Table S3 and Fig. S1*A* and *SI Methods*). The two-site model with binding cooperativity can only capture the stimulus-specific NFκB dependence when $h_{I}=3$ (*SI Appendix*, Fig. S1*B*) and binding cooperativity is positive (*SI Appendix*, Fig. S1*C*), without any dependence on $h_{N}$ value, suggesting that nonlinear IRF binding is essential to produce all stimulus-specific responses. To investigate how degrees of sigmoidal binding affected model fits, we tested a two-site model with binding cooperativity and an extended range of IRF Hill coefficients (*SI Appendix*, Table S3 and Fig. S1*D*). We found that increasing $h_{I}$ values improve fit to NFκBko and PolyIC-stimulated WT conditions, while increasing $h_{I}>2$ reduces fit to PolyIC-stimulated IRF3/7ko condition (*SI Appendix*, Fig. S1*E*). As before, optimal models all have positive binding cooperativity (*SI Appendix*, Fig. S1*F*). Because each condition shows a unique IFNβ response, we compared the worst-fitting points between all two-site models. We determined that the best two-site model has positive binding cooperativity and ultrasensitive IRF binding with $h_{I}=3$. Furthermore, any models with linear IRF binding have at least one point that is very poorly fit, while ultrasensitive NFκB binding does not noticeably affect any model fits (*SI Appendix*, Fig. S1*G*).

### A Three-Site Model Can Account for the Experimental Data.

**Fig. 2.**
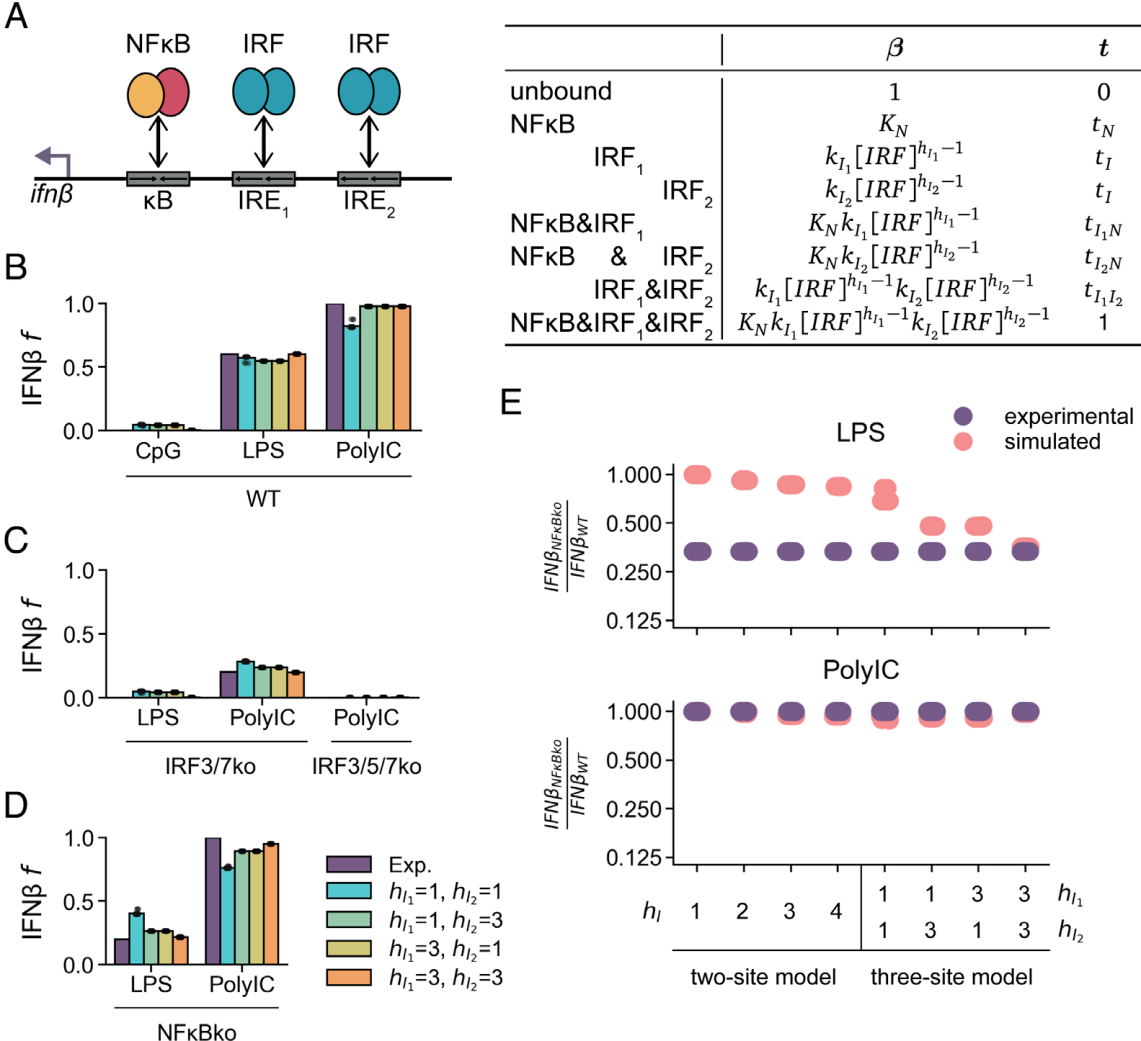
A three-site model accounts for the data in specific regulatory parameter regimes that involve nonlinear binding by IRFs. (*A*) *Left*, schematic of three-site thermodynamic state model, with two IRE sites and one κB site. Each protein has a corresponding binding affinity and activity. *Right*, table of vectors for binding equilibrium constants (β) and transcriptional activity (t) for each state of the enhancer. (*B*–*D*) Experimental and simulated IFNβ expression for four models of $h_{I_{1}}$ and $h_{I_{2}}$, in (B) WT, (*C*) IRF-dependent conditions, (*D*) NFκB-dependent conditions. Dots show predicted expression of best 20 optimized parameter sets, and bars show mean. (*E*) Ratio of experimental and simulated IFNβ expression in NFκBko over WT for LPS and PolyIC stimulus for all two-site and three-site models from best 20 optimized parameter sets.

For a more realistic model of the enhancer, we next tested a three-site model (*SI Appendix*, Fig. S2*A* and Table S4), comprising one binding site for NFκB, a proximal IRF binding site (IRE_1_) and a distal IRF binding site (IRE_2_). IRE_1_, IRE_2_, and NFκB have binding equilibriums of $k_{I_{1}}{\left[IRF\right]}^{h_{I_{1}}-1}$, $k_{I_{2}}{\left[IRF\right]}^{h_{I_{2}}-1}$, and $k_{N}{\left[NF\kappa B\right]}^{h_{N}-1}$ for their respective bound IRFs, where each k is a binding parameter and each h is a Hill coefficient that determines the linearity of binding to the cognate DNA sequence. This model has eight states of binding: unbound, NFκB bound, IRF bound to IRE_1_ (IRF_1_), IRF bound to IRE_2_ (IRF_2_), NFκB&IRF_1_, NFκB&IRF_2_, IRF_1_&IRF_2_, and NFκB&IRF_1_&IRF_2_. The unbound and fully bound states are assumed to have transcriptional activities of 0 and 1, respectively, while the transcriptional activities for the NFκB alone state ($t_{N}$) and the IRF alone states ($t_{I}$) are both unknown. New parameters are also defined for transcription from the states with two sites bound: $t_{I_{1}N}$ for the NFκB&IRF_1_ state, $t_{I_{2}N}$ for the NFκB&IRF_2_ state, and $t_{I_{1}I_{2}}$ for the IRF_1_&IRF_2_ state. These new parameters allow for functional synergy (e.g., $t_{I_{1}N}>t_{I_{1}}+t_{N}$) or antagonism (e.g., $t_{I_{1}N}<t_{I_{1}}+t_{N}$) between the different TFs, even in the absence of binding cooperativity. As the two-site model required ultrasensitive IRF binding but not ultrasensitive NFκB binding, we first tested four models in which none or one of the three dimers had ultrasensitive binding (*SI Appendix*, Fig. S2*A*). We found that the model where NFκB has ultrasensitive binding was no different from the model where NFκB has linear binding (*SI Appendix*, Fig. S2*B*), even when comparing all combinations of ultrasensitive/linear binding (*SI Appendix*, Fig. S2*C*). Consequently, for the subsequent analyses, we fixed NFκB to have linear binding described by the affinity parameter $K_{N}$ (Fig. 2*A*).

We identified optimized parameter sets for four different models, where ultrasensitive binding applies to neither IRF ($h_{I_{1}}=1,h_{I_{2}}=1$; i.e., the 1&1 model), only the distal IRF (the 1&3 model), only the proximal IRF (the 3&1 model), or both (the 3&3 model). All models adequately fit the data of stimulus-specific expression in wild type cells, though a high Hill coefficient for either IRF site improves the fit to PolyIC (Fig. 2*B*). All models correctly predict deficiencies in IRFko cells, with a high Hill coefficient for both IRF sites (the 3&3 model) improving the fit (Fig. 2*C*). Only the 1&3, 3&1, and 3&3 models closely recapitulate the NFκB dependence found in the literature (Fig. 2*D*), showing a decrease in IFNβ in the NFκBko compared to WT under LPS stimulus not seen in the 1&1 model or any two-site model without binding cooperativity (Fig. 2*E*). We concluded that the three-site state ensemble model with nonlinear binding of IRFs to IREs sufficiently accounts for the available IFNβ expression data.

We similarly fit three-site models with binding cooperativity between NFκB and IRF (*SI Appendix*, Table S5 and Fig. S3*A* and *SI Methods*). Unlike with the two-site model, allowing binding cooperativity did not lead to substantially improved fits to the data (*SI Appendix*, Fig. S3*B*), though all optimized models showed positive binding cooperativity (*SI Appendix*, Fig. S3*C*). As before, we compared the worst-fitting conditions in all three-site models. Any 1&1 model was unable to fit the data, even with binding cooperativity, and the 3&3 models showed the best fit (*SI Appendix*, Fig. S3*D*).

### p50-Homodimer Tunes the NFκB-IRF Regulatory Logic.

**Fig. 3.**
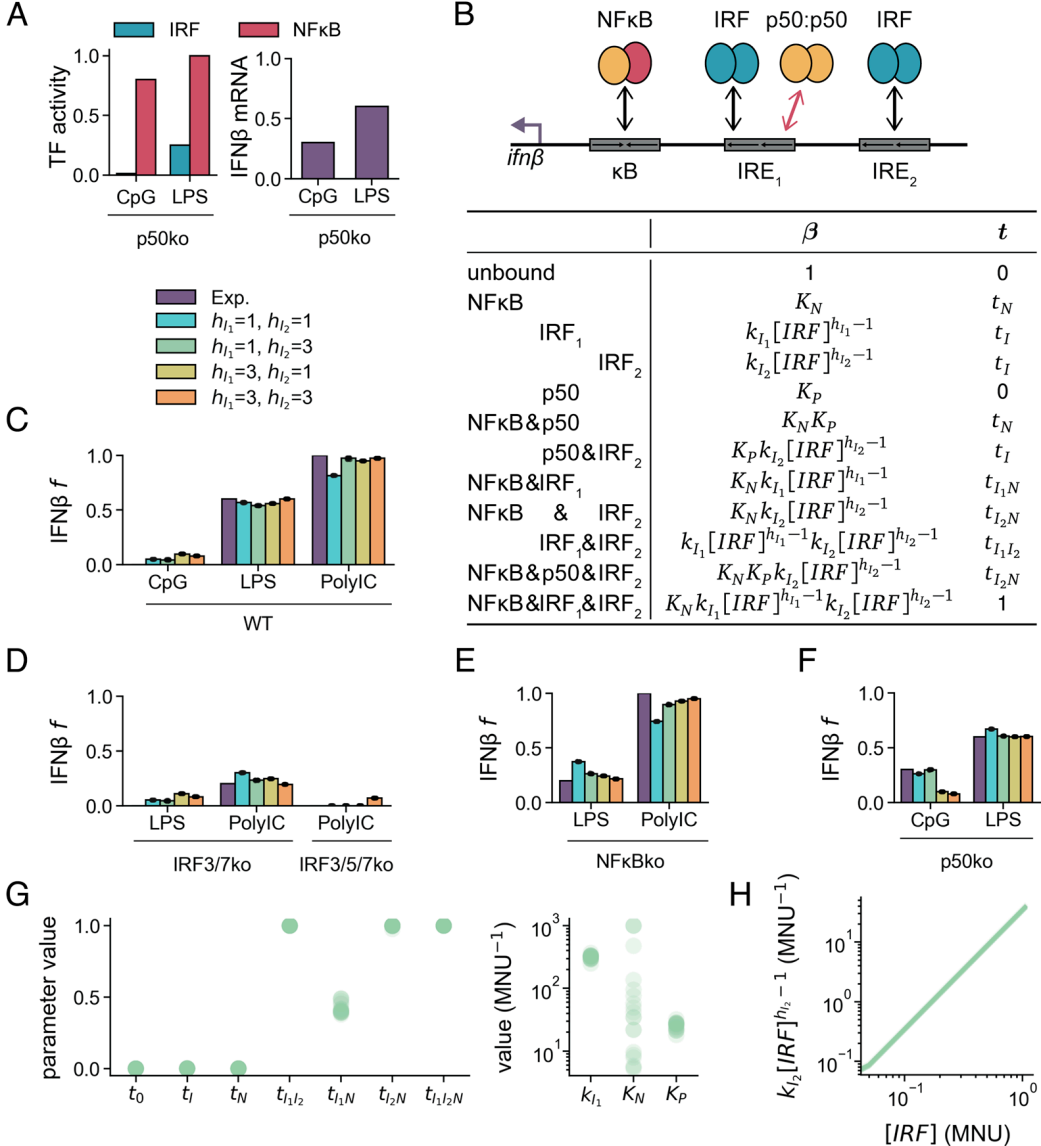
IRF responsiveness of IRE_1_ is tuned by the NFκB p50 homodimer. (*A*) Experimental data showing MNU of NFκB and IRF and max-normalized expression of IFNβ mRNA in p50ko conditions. (*B*) Above, schematic of the three-site thermodynamic state model with competitive inhibition of IRF by p50:p50 homodimer at the proximal IRE site. Each protein has a corresponding binding affinity and activity. Below, table of vectors for binding equilibrium constants (β) and transcriptional activity (t) for each state of the enhancer. (*C*–*F*) Experimental and simulated IFNβ expression for four models of $h_{I_{1}}$ and $h_{I_{2}}$ in (*C*) WT, (*D*) IRF-knockout conditions, (*E*) NFκB-knockout conditions, (*F*) p50:p50-knockout conditions. Dots show predicted expression of best 20 optimized parameter sets, and bars show mean. (*G*) Best 20 optimized t, $k_{I_{1}}$, $K_{N}$, and $K_{P}$ parameter values for all data points in $h_{I_{1}}=1,h_{I_{2}}=3$ model. (*H*) Best 20 optimized IRF_2_ binding equilibrium as a function of IRF concentration in $h_{I_{1}}=1,h_{I_{2}}=3$ model.

We next tested the three-site model with the previously reported finding that the p50:p50 homodimer binds the IRE_1_ binding site, competitively inhibiting IRF binding and IFNβ expression (30). In the absence of p50:p50, CpG, which does not activate IRF, is able to induce substantial IFNβ expression. However, the deficiency in p50:p50 results in only a minor increase in IFNβ with LPS (30). We added the p50ko condition to our collection of data (Fig. 3*A*).

We extended the three-site model to include p50:p50 vs. IRF competition for IRE_1_ (Fig. 3*B* and *SI Appendix*, Table S6). p50:p50 may bind at the same time as IRF_2_ and/or NFκB, but not IRF_1_, resulting in four additional enhancer binding states. p50:p50 does not contribute to transcription, such that transcription is determined only by any other bound TFs. We introduced the binding affinity of p50:p50 for IRE_1_ ($K_{P}$) as a free parameter to be determined by the available experimental data. We fit parameters for four models with linear NFκB binding and linear or ultrasensitive IRF binding (1&1, 1&3, 3&1, 3&3) and selected the top 20 optimized parameter sets. All models fit the WT data, though the 1&1 model has a worse fit to the WT PolyIC-stimulated condition (Fig. 3*C*). IRF-dependent conditions are fit well across models, especially in the 1&3 and 3&1 models (Fig. 3*D*). Again, the 1&1 model did not exhibit as much stimulus-specific NFκB dependence as the other models (Fig. 3*E*). In the p50ko condition, while LPS expression was well fit across all models, CpG expression was only fit by the 1&1 and 1&3 models (Fig. 3*F*). Consequently, we proceeded with the 1&3 model, as it satisfactorily captured all observations.

The optimized parameters demonstrate high levels of functional synergy, where $t_{I}$ and $t_{N}$ are approximately 0 and $t_{I_{1}I_{2}}$ and $t_{I_{2}N}$ are approximately 1, and $t_{I_{1}N}$ is around 0.5 (Fig. 3 *G*, *Left*). Consequently, five of the twelve enhancer states (NFκB&IRF_1_, NFκB&IRF_2_, IRF_1_&IRF_2_, NFκB&p50&IRF_2_, and NFκB&IRF_1_&IRF_2_) are transcriptionally active, four of which (INFκB&IRF_2_, IRF_1_&IRF_2_, NFκB&p50&IRF_2_, and NFκB&IRF_1_&IRF_2_) are maximally active.

We next examined binding affinity in the 1&3 model. $k_{I_{1}}$ is an order of magnitude higher than $K_{P}$ (≈300 MNU^−1^ compared to ≈25 MNU^−1^), which may be crucial for IRF to compete with p50:p50 when sufficient IRF is stimulated (Fig. 3 *G*, *Right*). The maximal binding affinity for IRF_2_ ($k_{I_{2}}{\left[IRF\right]}^{h_{2}-1}$), when $\left[IRF\right]=1$ MNU, is ≈ 30 MNU^−1^, also lower than $k_{I_{1}}$ (Fig. 3*H*). At lower IRF concentrations it is substantially lower. $K_{N}$ is not well-determined by the data, as a range of values from 5 to 10^3^ MNU^−1^ satisfy the observations (Fig. 3 *G*, *Right*). We concluded that the increased dynamic range of the IRF_2_ binding affinity is essential for capturing the high values of IFNβ in PolyIC-stimulated WT and NFκBko conditions. We conclude that a model where IRF_1_ has a binding affinity high enough to compete with p50:p50, IRF_2_ has sigmoidal binding, and all TF pairs have high synergy can explain all data.

### Alternate Model Conceptions Show No Improvement.

We then investigated whether binding cooperativity would improve the fit of the model. We first added a parameter to allow positive or negative binding cooperativity between IRF dimers at IRE_1_ and IRE_2_ (*SI Appendix*, Table S7 and Fig. S4*A* and *SI Methods*). Positive cooperativity between IRF dimers slightly improved the fit of the 1&3 model, which remained the only acceptable model (*SI Appendix*, Fig. S4 *B* and *C*). We next added a parameter to the three-site model with p50:p50 competition to allow binding cooperativity between NFκB and either IRF (*SI Appendix*, Table S8 and Fig. S4*D*). Similarly, the 1&3 model remained the only acceptable model even with positive cooperativity between IRF and NFκB (*SI Appendix*, Fig. S4 *E* and *F*). A comparison of the worst-fitting conditions among all three-site models with p50:p50 competition confirmed that ultrasensitive binding of the distal IRF and linear binding of the proximal IRF is essential for describing all data points, while binding cooperativity between IRF dimers or between IRF and NFκB leads to only minor improvements, and linear NFκB binding is sufficient (*SI Appendix*, Fig. S4*G*). We concluded that binding cooperativity between IRF and NFκB is dispensable for fitting the available data.

Next, we considered that functional synergy may plausibly be only possible between neighboring proteins and tested a model with $t_{I_{2}N}=t_{I_{2}}+t_{N}$ (*SI Appendix*, Table S9 and Fig. S5*A* and *SI Methods*). We found that again only the 1&3 model was acceptable (*SI Appendix*, Fig. S5*B*). With distal synergy disallowed, $t_{I_{1}N}$ is approximately 1, so all states yield either no transcription or maximal transcription (*SI Appendix*, Fig. S5*C*). As before, $k_{I_{1}}>K_{P}$ and $k_{I_{1}}>k_{I_{2}}$ for all IRF activities (*SI Appendix*, Fig. S5*D*). Therefore, the only difference in this model is a higher $t_{I_{1}N}$.

### Modes of Synergy Explain Stimulus Specificities.

**Fig. 4.**
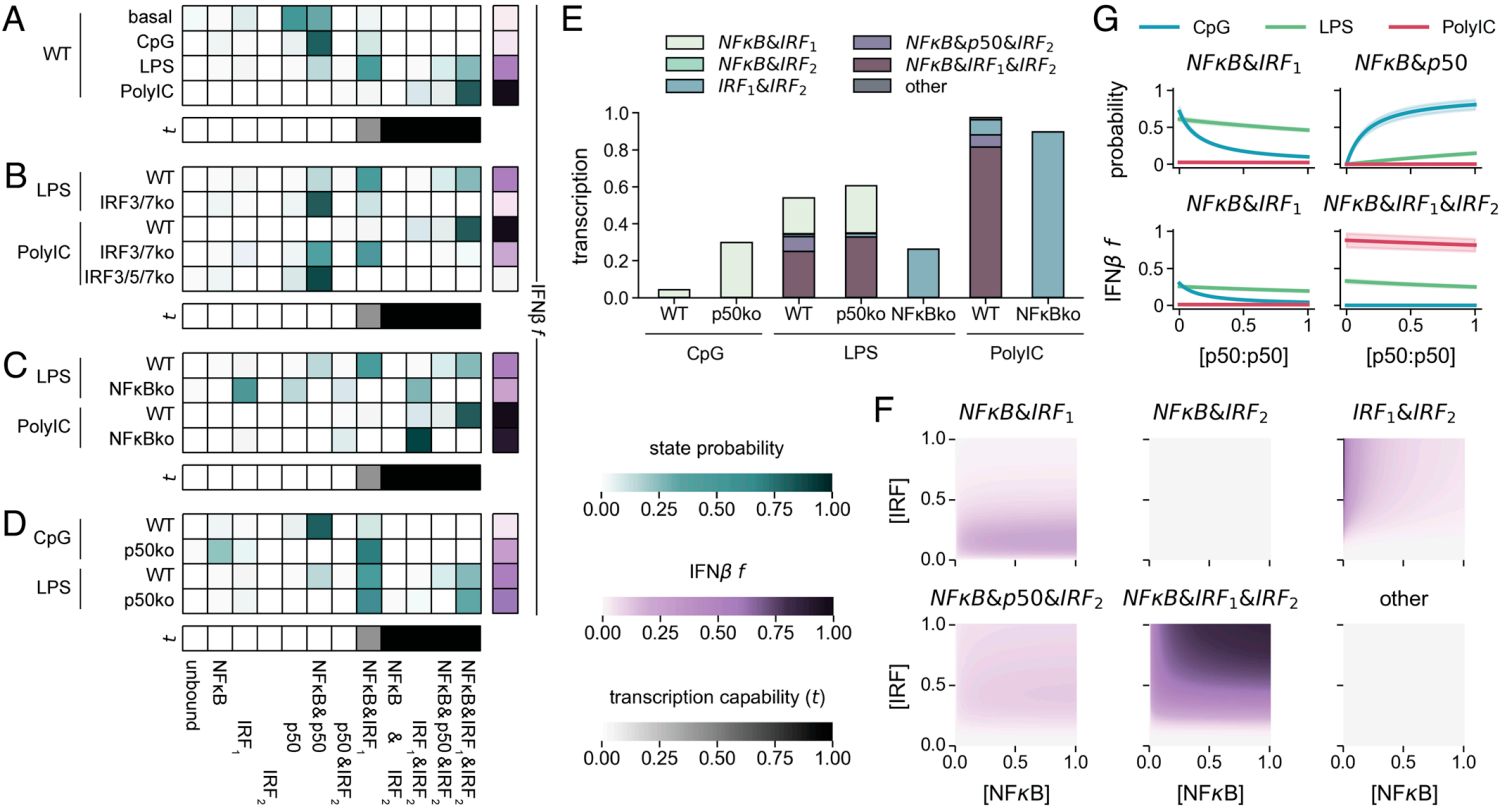
The enhancer state model reveals regulatory modes and can be used for forward predictions. (*A*–*D*) Heatmaps of the state probabilities (blue color bar) determined by the best-fit model. Bottom row (brown color bar) shows a heatmap of transcription capability (t) parameter for each state. Column on right (purple color bar) shows a heatmap of IFNβ f from model fit. State probability and f values are shown for: (*A*) WT in unstimulated and stimulated conditions, (*B*) IRF-deficient and corresponding WT conditions, (*C*) NFκB-deficient and corresponding WT conditions, (*D*) p50-deficient and corresponding WT conditions. (*E*) Bar plot of amount of transcription contributed by each state in stimulus-specific NFκB- and p50-dependent conditions determined by the best-fit model. (*F*) Heatmaps of the amount of transcription coming from each state for a range of NFκB and IRF input concentrations in WT predicted by the best-fit model. (*G*) Mean and SD of predicted probability of the NFκB&IRF_1_ and NFκB&p50 states (*Top*) and IFNβ promoter activity due to the NFκB&IRF_1_ and NFκB&IRF_1_&IRF_2_ states (*Bottom*) for different concentrations of p50:p50 under CpG, LPS, and PolyIC stimulus.

Using the best-fitting 1&3 model, we investigated the probabilities of the 12 enhancer states under the 10 stimulus conditions (Fig. 4 *A*–*D*). In the inactive basal condition, the enhancer is primarily in the inactive p50 (54%) and NFκB&p50 (32%) states (Fig. 4*A*), while upon CpG stimulation the NFκB&p50 state is dominant (81%) and the probability of being in an active state remains low (10%). However, upon LPS stimulation, the probabilities of enhancer states are 25% for the highly active NFκB&IRF_1_&IRF_2_ state, 46% for the partially active NFκB&IRF_1_ state, and only 15% for the inactive NFκB&p50 state. In response to PolyIC, the enhancer is almost entirely in the NFκB&IRF_1_&IRF_2_ state (82%). The differences in active state probabilities explain the stimulus-specificity of IFNβ expression, where CpG stimulates little, PolyI:C a lot, and LPS a moderate amount of expression.

Reducing IRF activity perturbs the state distributions. In LPS-stimulated IRF3/7ko cells, the probability of the NFκB&IRF_1_ state decreases to 10%, leaving 82% for the NFκB&p50 state, explaining why almost all transcription is lost in this condition (Fig. 4*B*). When the IRF3/7ko is stimulated with PolyIC, the partially active NFκB&IRF_1_ state is accessed (48%), allowing the retention of some IFNβ production. This is due to the high $k_{I_{1}}$ allowing even low concentrations of IRF to effectively compete with basally bound p50:p50. However, when all IRF activity is ablated in IRF3/5/7ko cells, the enhancer almost entirely occupies the inactive NFκB&p50 state (87%).

In LPS-stimulated NFκBko cells, the enhancer shifts not only from the NFκB&IRF_1_&IRF_2_ state (25%) to the equally active IRF_1_&IRF_2_ state (26%) but also from the active NFκB&IRF_1_ state (46%) into the inactive IRF_1_ state (49%), resulting in a decrease in IFNβ transcription (Fig. 4*C*). Because PolyIC-stimulated WT cells specify primarily the NFκB&IRF_1_&IRF_2_ state, NFκB deficiency results in the equally active IRF_1_&IRF_2_ state (90%), explaining the NFκB-independence in this condition.

While CpG does not activate IRF, in p50ko cells, basal IRF binding to the high affinity IRE_1_ and CpG-induced NFκB are sufficient for the enhancer to have a high probability (71%) of occupying the partially active NFκB&IRF_1_ state (Fig. 4*D*). However, in response to LPS, the probabilities of accessing the NFκB&IRF_1_ or NFκB&IRF_1_&IRF_2_ states only increase moderately over the WT condition (61 vs. 46% and 33 vs. 25%, respectively) given that LPS-induced IRF is sufficient to overcome the p50:p50 blockade in WT cells. This moderate increase in p50ko cells is associated with the loss of the IRF_2_&NFκB&p50 state.

We also calculated the probability of each active state across all knockout conditions for each stimulus (*SI Appendix*, Fig. S5*E*). Under CpG stimulus, the only activate enhancer state is the NFκB&IRF_1_ state, but this is limited by the low basal level of IRF and the competition from p50:p50. Under LPS or PolyIC stimulus, the enhancer can occupy all active states, though its probability of being in the NFκB&IRF_2_ state is only 0.2% or 0.1%, respectively, and its probability of being in the NFκB&p50&IRF_2_ state is only 2.0% for each stimulus. As these states are rarely accessed, we concluded that their contribution to IFNβ transcription is negligible.

To further understand the contribution of each state to IFNβ transcription in each condition, we examined the amount of max-normalized transcription units (TUs) contributed by each state (Fig. 4*E*). In response to CpG, all transcription comes from the NFκB&IRF_1_ state (0.04 TU), which increases in p50ko cells (0.30 TU).

In response to LPS, transcription comes from NFκB&IRF_1_&IRF_2_ (0.25 TU), NFκB&IRF_1_ (0.19 TU), and IRF_1_&IRF_2_ (0.01 TU) states. Transcription from each of these states increases moderately when p50:p50 is absent (to 0.33, 0.26, and 0.02 TU, respectively), given that the NFκB&p50&IRF_2_ state is eliminated. In NFκBko cells, the only available active state is IRF_1_&IRF_2_ (Fig. 4*E*). In response to LPS, transcription from this state increases (from 0.01 to 0.26 TUs), reflecting the sum of the transcription from the IRF_1_&IRF_2_ and the NFκB&IRF_1_&IRF_2_ states in WT. However, this does not fully compensate for the loss of the NFκB&IRF_1_ state (from 0.19 TU to 0 TUs), causing an overall decrease in transcription.

In response to PolyIC, most transcription is from the IRF_1_&IRF_2_ (0.08 TU) and the NFκB&IRF_1_&IRF_2_ (0.81 TU) states, which converges on the IRF_1_&IRF_2_ state in the absence of NFκB (0.90 TU). All other active states show only a negligible loss of transcription in the NFκBko (0.08 TU). Overall, the probability distributions of active states explain the stimulus-specific and knockout condition-specific transcriptional control of IFNβ.

### Using the Model for Forward Predictions.

To use the combinatorial states model for forward predictions of biological scenarios not directly represented by the training data, we first asked whether the primary conclusions and best-fit parameters were robust to variations in the training data due to uncertainties in measurements. We generated 100 synthetic datasets with different levels of noise added to the NFκB and IRF input values and optimized parameters as before to the synthetic datasets (*SI Appendix*, *SI Methods* and Fig. S6 *A*–*D*). When noise corresponded to 1% error, best fit t parameters matched those in the original data, and best fit k parameters followed the same patterns as the original with a slightly wider range of values (*SI Appendix*, Fig. S6*E*). With 10% (*SI Appendix*, Fig. S6*F*) and 20% (*SI Appendix*, Fig. S6*G*) error, all best fit t parameters continued to match the original data except for $t_{I_{1}N}$, which had an increased upward spread toward 1. Only with 40% error did $t_{I_{2}N}$ and $t_{I_{1}N}$ have high variation (*SI Appendix*, Fig. S6*H*). Importantly, we found that even at 20% noise, synergy was still required between the two IRFs as well as each IRF and NFκB (i.e., at least two sites must be bound to initiate transcription, *SI Appendix*, Fig. S6*G*). We conclude that $t_{I_{1}N}$ is the least robust parameter and that the synergy requirement of NFκB&IRF_1_ may be less robust than that of IRF_1_&IRF_2_ or IRF_2_&NFκB, but that NFκB is always dispensable when IRF_1_ and IRF_2_ are bound.

We wondered whether underlying noise might lead to poor fits from models that could otherwise fit the data. However, even when allowing 40% error in the original data, the 1&1 model is unable to fit the NFκBko conditions, and the 3&1 and 3&3 models are unable to fit the p50ko conditions (*SI Appendix*, Fig. S6*I*), confirming that the 1&3 model is the only possible description of IFNβ regulation. These studies indicated that our conclusions are robust to error in the assembled data matrix, providing confidence in the reliability of forward predictions in conditions outside of the training dataset.

We next undertook comprehensive dose–response studies, predicting IFNβ transcription for a range of NFκB and IRF activities (Fig. 4*F*). With enough IRF (>0.2 MNU) and nonzero NFκB, the NFκB&IRF_1_&IRF_2_ state produces a large amount of IFNβ transcription and the NFκB&p50&IRF_2_ state contributes to a much smaller extent. With enough IRF and low NFκB, the IRF_1_&IRF_2_ state produces moderate amounts of IFNβ transcription. With low but nonzero IRF, the NFκB&IRF_1_ state produces most IFNβ transcription. All other states contribute very little to IFNβ transcription under any NFκB and IRF activities. We also predicted IFNβ transcription in p50ko cells and found that the lower the concentration of IRF, the stronger the repressive effect of p50:p50 (*SI Appendix*, Fig. S4*J*). We further explored stimulus specificity as a function of p50:p50, finding that increased [p50:p50] decreased the probability of formation and transcription from the NFκB&IRF_1_ state and increased the probability of the NFκB&p50 state under CpG and LPS stimulus while mildly decreasing transcription from the NFκB&IRF_1_&IRF_2_ state under LPS and PolyIC stimulus (Fig. 4*G*). These analyses reveal how IFNβ transcription can be tuned by different stimuli and cellular conditions that involve different IRF and NFκB activity levels.

**Fig. 5.**
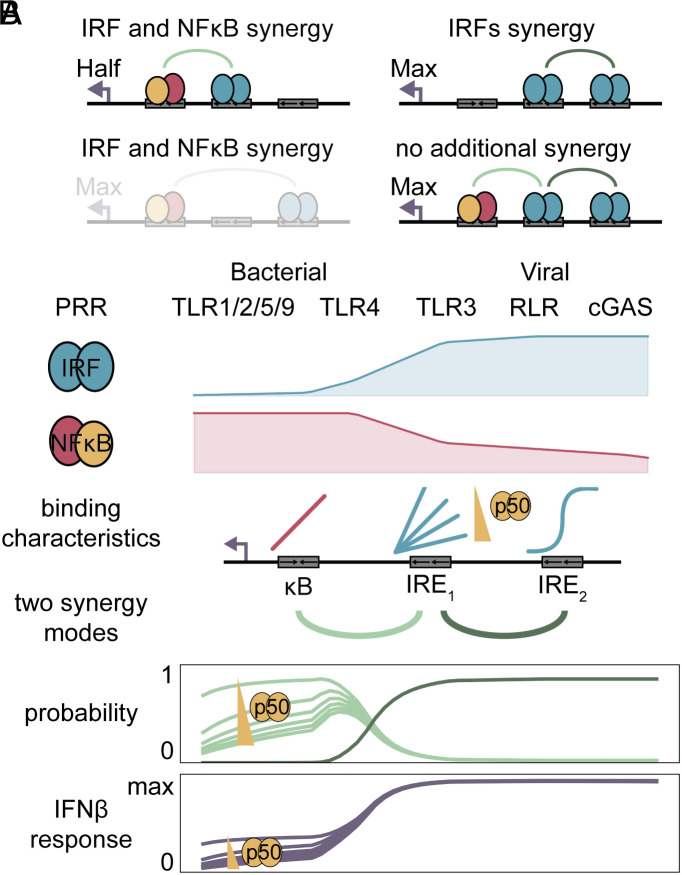
The enhancer state model differentially uses two regulatory modes. (*A*) Schematic summarizing synergy modes of the IFNβ enhancer. The NFκB&IRF_1_ state (*Upper Left*) shows synergy between IRF and NFκB leading to half of maximal transcription. The IRF_1_&IRF_2_ state (*Upper Right*) shows synergy between IRFs leading to maximal transcription. The NFκB&IRF_2_ state (*Lower Left*) may also be synergistic but is rarely used. The NFκB&IRF_1_&IRF_2_ state (*Lower Right*) has no additional synergy beyond the maximal transcription provided by aforementioned synergy modes. (*B*) Summary of the stimulus-specific usage of the two synergy modes. Depending on which pattern recognition receptor (PRR) is stimulated, different levels of IRF and NFκB activities are activated. The TFs bind to the IFNβ enhancer such that the proximal IRE is tunable by competition with the p50 homodimer and the distal IRE shows nonlinear binding. Two distinct synergy modes have distinct characteristics: The first is tunable by p50, and the second is robust but requires high IRF activity. As a result, IFNβ is reliably expressed in response to viral PAMPs, but its expression in response to bacterial exposure depends on the precise PAMP and the context of exposure history, which can determine p50 levels.

Our study identifies two synergy modes controlling transcription of IFNβ: synergy between NFκB and the proximal IRF, and synergy between two IRFs (Fig. 5*A*). Synergy between NFκB and the distal IRF is rarely used, and the fully bound enhancer has no additional synergy beyond these two modes. The two synergy modes are differentially accessed to respond to specific PAMPs (Fig. 5*B*). PRRs leading to low IRF and high NFκB predominantly yield IFNβ in the NFκB and IRF synergy mode. This mode has decreasing probability with increasing p50:p50 concentrations by competing with IRF for binding at IRE_1_. As a result, IFNβ expression in response to these PRRs is tunable by modulating p50:p50 concentrations, which are a function of prior exposure history to inflammatory stimuli (46). In contrast, PRRs leading to high IRF activation predominantly yield IFNβ via the IRFs synergy mode as the high IRF concentration overcomes the thresholded IRE_2_ binding curve and is unaffected by potential competition from p50:p50 at IRE_1_. Thus, IFNβ expression in response to viral PAMPs is more robust, while it is more tunable in response to bacterial PAMPs.

## Discussion

We report here the development of a quantitative model of the regulatory logic that governs IFNβ expression. It recapitulates a variety of stimulus–response data exploring the stimulus-specificity of IFNβ expression, its dependence on IRF and NFκB activators, and the role of the repressor p50:p50. We tested alternate models that are both qualitatively and quantitatively distinct in how the three transcription factors function together, synergistically or competitively, to control IFNβ expression. Even with limited data, we rejected many different model formulations, including various two-site and three-site models, and converged on specific regulatory insights. The resulting model reveals that each binding site and TF has a nonredundant regulatory role that is governed by quantitative specifications of its dose–response curve as well as functional interactions with partnering TFs on the enhancer. The specific role of each binding site explains why the enhancer sequence is so highly evolutionarily conserved and has no single nucleotide polymorphisms with a prevalence above 0.03% on dbSNP (47). The model indicates two modes of synergy responsible for the majority of stimulus-specific IFNβ expression. Furthermore, the model had highly constrained parameters for IRF and p50:p50 binding and for transcription capability of each state that were robust to potential measurement error.

We found a higher binding affinity for IRE_1_ than for IRE_2_ in all acceptable models. This is in line with biochemical studies, which have noted that the first half-site of IRE_1_ is GAGA, while IRE_2_ contains the suboptimal AAAA (43, 48). Additionally, protein-binding microarrays have found that IRF dimers bind more strongly to the IRE_1_ sequence despite its suboptimal 3 bp spacing between half-sites (48, 49). The model also required a nonlinear ultrasensitive dose–response curve (i.e. a Hill coefficient above 1) at IRE_2_, but not at IRE_1_, where basal p50:p50 prevents spurious IRF binding. We note that IRF at IRE_2_ may interact with neighboring and constitutively present AP1 (50), which could result in multivalent, nonlinear binding. This remarkable agreement between the model inferred from stimulus–response data and biochemical findings not used for model fitting provides confidence in the model and offers support for the functional relevance of prior biochemical studies.

Given that all known IRF3-inducing stimuli also activate NFκB, NFκB is active in every WT pathogen-response condition. Each of these conditions yield NFκB bound to the enhancer, but whether NFκB binding is functionally important depends on context. Alone, it is unable to drive transcription of IFNβ, and when both IRF_1_ and IRF_2_ are present it is dispensable. Therefore, NFκB plays a functional role only in conditions of low IRF activation. Given that IRF has a higher binding affinity for IRE_1_ than for IRE_2_, in the conditions tested here NFκB rarely synergizes with IRF bound to the distal site, but we may speculate that in conditions of very high p50:p50 homodimer, NFκB might functionally synergize with the distal IRE_2_ to activate IFNβ expression. In such a condition, IFNβ expression would still occur, as NFκB is activated by all viral PAMPs. In contrast, IFNβ is tunable in response to bacterial PAMPs that do not or barely activate IRF3, such as Pam3CSK, flagellin, or CpG, and even LPS. As the *nfkb1* gene is NFκB-inducible (51) and p50 is generated by processing of p105 via IKK2 (46) it constitutes a feedforward loop (52) that has the potential to maintain innate immune memory: Cells pre-exposed to stimuli can contain higher levels of p50:p50 than naïve cells, reducing the contribution of the NFκB&IRF_1_ to IFNβ expression.

Previous research did not fully delineate the molecular mechanisms that mediate transcription factor synergy. The simplest explanation that IRF and NFκB have cooperativity in binding was distinguished from functional synergy by the present study and found not to make essential contributions. Prior work noted that the two bound IRFs and NFκB do not have direct contacts (43, 48, 50) and NFκB and the proximal IRF have no binding cooperativity when measured by EMSA (43). Still, cooperative binding could be mediated by CBP/p300 contacts (44, 45, 53). CBP has disordered, flexible regions (54) and can adapt to different distances between the binding domains of IFNβ (43). However, we show here that binding cooperativity is insufficient to explain observed synergy. Instead, synergy may come from a subsequent mechanistic step, perhaps involving activation of the histone acetyltransferase function of CBP/p300 by IRF (55). An alternate mechanism for synergy may be kinetic in which different regulatory steps of preinitiation complex formation, transcriptional initiation, and elongation are determined by different TFs, but given that two IRFs are sufficient, such an underlying mechanism is unlikely.

Extending the model in the future can address different biological scenarios. Our model reflects pathogen sensing via PRR; future work could consider the regulation of IFNβ half-life (56), lower dependence on IRF3 and IRF7 (57), and roles of upstream enhancer elements (58) that occur during live infection. Data from additional biological conditions would not bring back models that were excluded by the present study, but it may add complexity to reveal intricacies of IFNβ regulation that are not yet captured. Similarly, the present model considers only IRF and NFκB as the key regulators of IFNβ expression, as early studies noted that AP1 and NFκB activation is always coordinated (59–62) and interdependent (63), and more recent studies described AP1’s function as a marker for open chromatin rather than a transcriptional activator (64, 65). However, additional data may necessitate the explicit consideration of AP1 in the regulatory logic of IFNβ expression.

The quantitative model of IFNβ regulation presented here contributes to 40 y of research into the regulation of the IFNβ enhancer. Though models of immune-response gene expression have advanced (52, 66–70), IFNβ has long been a bottleneck in the development of interferon response models (71, 72). The present model for IFNβ promoter activity may therefore be a building block for the development of more comprehensive models that connect pathogen response signaling pathways with the large ISG expression program, expanding their utility for studies of IFNβ-related diseases as well, as molecular mechanisms for many inborn IFNβ deficiencies have not yet been identified (10).

## Materials and Methods

Data were collected from a variety of published sources (30, 31, 32, 33, 34) to generate a data table for NFκB and IRF transcription factor activity in response to CpG, LPS, and polyIC, along with IFNβ gene expression in these conditions. These data were normalized between 0 and 1 to allow for cross-comparisons. Mathematical models were constructed using the Thermodynamic State Ensemble formalism (35, 36), in which each singly or combinatorially bound promoter state is enumerated. Each state is associated with a promoter activity or transcription parameter; transcriptional synergy is evident when the promoter activity of a combinatorially bound state is greater than the sum of the singly bound states. This formalism was used to construct two-site models of NFκB and IRF with binding Hill coefficients of 1 and 3, and with and without binding cooperativity between them. Then analogous versions of three-site models of two IRF and one NFκB binding sites were constructed. Additional states in which p50 is bound to the proximal IRF finding site were then introduced to model prior findings (30). For each model, parameters were sampled and then optimized using the Scipy v1.11.4 (73) implementation of the Nelder Mead algorithm. The robustness analysis involved generating 100 synthetic datasets containing sampled concentrations of NFκB and IRF for each of the 10 conditions with 1, 10, 20, or 40% error. Details of all methods are described in *SI Appendix*.

## Supplementary Material

Appendix 01 (PDF)

## Data Availability

Modeling code has been deposited in Github (https://github.com/signalingsystemslab/IFNb_Gene_Regulatory_Logic) (74). All other data are included in the manuscript and/or *SI Appendix*. Previously published data were used for this work (32).
